# Place of death in Parkinson's disease: A systematic review and meta-analysis of associated factors

**DOI:** 10.1177/1877718X261431105

**Published:** 2026-04-17

**Authors:** Anna J Pedrosa, Florian Kuschel, Hanna K Fischer, David J Pedrosa

**Affiliations:** 1 Marburg University, Faculty of Medicine, Department of Neurology, Marburg, Germany; 2 Marburg University, Centre for Mind, Brain and Behaviour, Marburg, Germany

**Keywords:** death, home care, hospitals, hospice care, hospices, nursing homes, Parkinson disease, palliative care, terminal care

## Abstract

**Introduction:**

Parkinson's disease is associated with increased mortality and hospitalisations are common at the end of life. However, limited evidence exists regarding the place of death and its influencing factors in people with Parkinson's disease (PwPD).

**Objectives:**

To identify and analyse factors associated with place of death in PwPD.

**Methods:**

We systematically searched three electronic databases (MEDLINE, EMBASE, PsycINFO) for studies reporting on the place of death of PwPD. No restrictions on time or language were applied. Where possible, meta-analyses were conducted using random-effects meta-regression adjusted for country-level long-term care and hospital bed availability. Results are presented as odds ratios (OR) for place of death with 95% confidence intervals. Sensitivity analyses were performed to explore heterogeneity.

**Results:**

33 studies were analysed, including over 1,200,000 individuals across five continents and reporting on individual, illness-level, service-level, and environmental factors. Hospital death was more likely among men (OR = 1.34; 95% CI: 1.21–1.49), married individuals (OR = 1.16; 95% CI: 1.07–1.26), and those under 85 years (OR = 1.29; 95% CI: 1.20–1.39). Lower-quality evidence suggested a higher likelihood of hospital death among non-white individuals, while receipt of palliative care was associated with reduced odds.

**Conclusions:**

This systematic review and meta-analysis identify key factors associated with hospital death in PwPD that can inform clinical decision-making and policy planning. Our findings may support the development of targeted screening interventions and help clinicians and policymakers allocate resources effectively. Further research is needed to address gaps in evidence across different care settings.

**Plain language title:**

Place of death in Parkinson's disease and related factors

## Plain language summary

People with Parkinson's disease have a higher risk of dying earlier, and many spend time in hospital towards the end of life. Yet, little is known about where people with Parkinson's typically die and what influences this.

We reviewed 33 studies from around the world, including data on more than 1.2 million people with Parkinson's disease. We looked at factors such as age, gender, marital status, and access to health care serviced to see how they affect place of death.

We found that hospital deaths were more common among men, married people, and those younger than 85 years. Some evidence suggested that people from minority ethnic groups were also more likely to die in hospital. In contrast, receiving palliative care reduced the probability of dying in hospital.

These findings highlight which groups may be at higher risk of hospital death. This knowledge can help healthcare providers and policymakers improve planning, target support, and develop services so that care at the end of life better reflects the needs and preferences of people with Parkinson's disease and their families.

## Introduction

Parkinson's disease (PD) is a common neurological condition.^1^ Overall, the global burden of PD expanded substantially, affecting an estimated 6.1 million individuals in 2016, and is anticipated to continue growing, driven by population aging, increased disease longevity, and possible environmental determinants.^2^ While motor manifestations remain the defining clinical features of Parkinson's disease, an extensive spectrum of non-motor symptoms, including psychological, cognitive, and autonomic disturbances, as well as social and spiritual factors, exerts an additional influence on patients’ quality of life and caregiver burden.^3–5^

People with Parkinson's disease (PwPD) experience higher rates of hospitalisations compared to the general population, a pattern that persists even at the final stages of life.^6,7^ While hospital admissions can serve as crisis interventions,^8^ they also lead to increased healthcare resource utilisation and, more importantly, disrupt continuity of care – an issue particularly detrimental for PwPD.^9–11^ Beyond disruptions in their care, several other reasons may negatively affect PwPD in the consequence of hospitalisations. Thus, hospital stays are often accompanied by medication errors, and the risk of hospital-acquired adverse events such as infections, confusion, falls, and decubitus.^12^ Furthermore, PwPD are more likely to die under hospital care than controls.^13^

Notably, many PwPD express a preference to receive end-of-life care and die in the comfort of their own home, as indicated by previous studies.^14–16^ However, a meta-analysis has demonstrated a significant and increasing association between non-cancer diagnoses and a mismatch between preferred and actual place of death.^17^ In this context, the high rate of end-of-life hospital admissions in PD highlights a potential failure to align care with patient values,^18^ raising concerns about uptake of advance care planning and access to palliative services,^19,20^ and the ability of family caregivers to support preferences for place of death.^21^ Place of death is therefore not only a reflection of individual preferences but also a widely recognised indicator of the quality, coordination and responsiveness of healthcare systems. Therefore, the discrepancy between these preferences and the reality of frequent end-of-life hospital admissions in PD underscores the need to better understand the factors influencing place of death in PwPD.

Although individual studies have explored facilitators and barriers related to place of death, a comprehensive and systematic synthesis of this evidence is currently lacking. To address this gap, our objective was to systematically identify and analyse the factors associated with place of death in PwPD. Specifically, this systematic review and meta-analysis sought to examine links between place of death and individual, illness-level, service-level, and environmental factors. By consolidating the available evidence, this review is expected to inform clinical practice, shape healthcare policy, and guide future research – all with the overarching goal of enhancing the quality of end-of-life care and support for PwPD.

## Methods

### Design

Using the PICO framework, the research question for this systematic review and meta-analysis was defined as follows: in people with Parkinson's disease (Population), which factors (Exposure) are associated with different places of death (Outcome). Accordingly, the primary outcome was the identification of factors associated with specific places of death. As a secondary outcome, we examined the distribution of place of death. The review protocol was not registered or published in advance. This systematic review is reported in accordance with the Preferred Reporting Items for Systematic Reviews and Meta-Analyses (PRISMA) guideline.^22^

### Eligibility criteria

We conducted a systematic literature review of studies that reported original data examining place of death and/or at least one factor associated with place of death in PwPD. Studies investigating in-hospital mortality only were excluded. Experimental, quasi-experimental, and observational study designs were considered for inclusion. Case reports, case series, reviews, and qualitative studies were excluded. A minimum number of ten deceased study participants was required for inclusion. Unpublished studies (i.e., conference abstracts, dissertations, and presentations) were included if they met the study design criteria. Studies reporting on mixed populations were considered if the results for the subpopulation with PwPD were reported separately or accounted for at least 50% of the population.

### Search strategy and study selection process

Three electronic databases (MEDLINE, EMBASE, PsycINFO) were searched in September 2023, using a combination of title/abstract keywords and MESH terms (cf. Supplement). The search was updated in April 2025. No language or time restrictions were applied. Citation tracking and reference searching of included studies were performed. Following import into EndNote 20, double screening was performed by AP and HF for all titles and abstracts to select eligible studies. Full text was retrieved if there was uncertainty about eligibility. Disagreements were resolved via discussions.

### Data collection process

For each included study, detailed information was extracted by AP or FK using a standardised data form including first author, year of publication, country of origin, population characteristics, sample size, factors studied, statistical analysis, results, and quality score.

### Quality assessment and strength of evidence

Double quality assessment was performed independently by AP and FK or HF using the validated Standard Quality Assessment Criteria for Evaluating Primary Research Papers from a Variety of Fields (QualSyst) tool.^23^

### Synthesis of results

We extracted or calculated odds ratios for factors associated with place of death as well as 95% confidence levels and levels of significance. Factors were grouped if feasible (e.g., combining different palliative care services). Results were synthesised by meta-analysis for each individual place of death if at least three studies investigated the same factor. National-level data were analysed separately to account for variability in healthcare systems, cultural norms, and data availability across the included countries. We used random-effects models for meta-analysis and performed a meta-regression model to adapt for country-specific availability of hospital and long-term care beds.^24,25^ We used funnel plots to assess for publication bias (cf. Supplement). Where meta-analysis was not possible, the results were summarised narratively. All analyses were performed using R (version 4.3.3)^26^ and the metafor package (version 4.6-0)^27^ and can be found under https://github.com/dpedrosac/POD_systreview.

## Results

**Figure 1.**
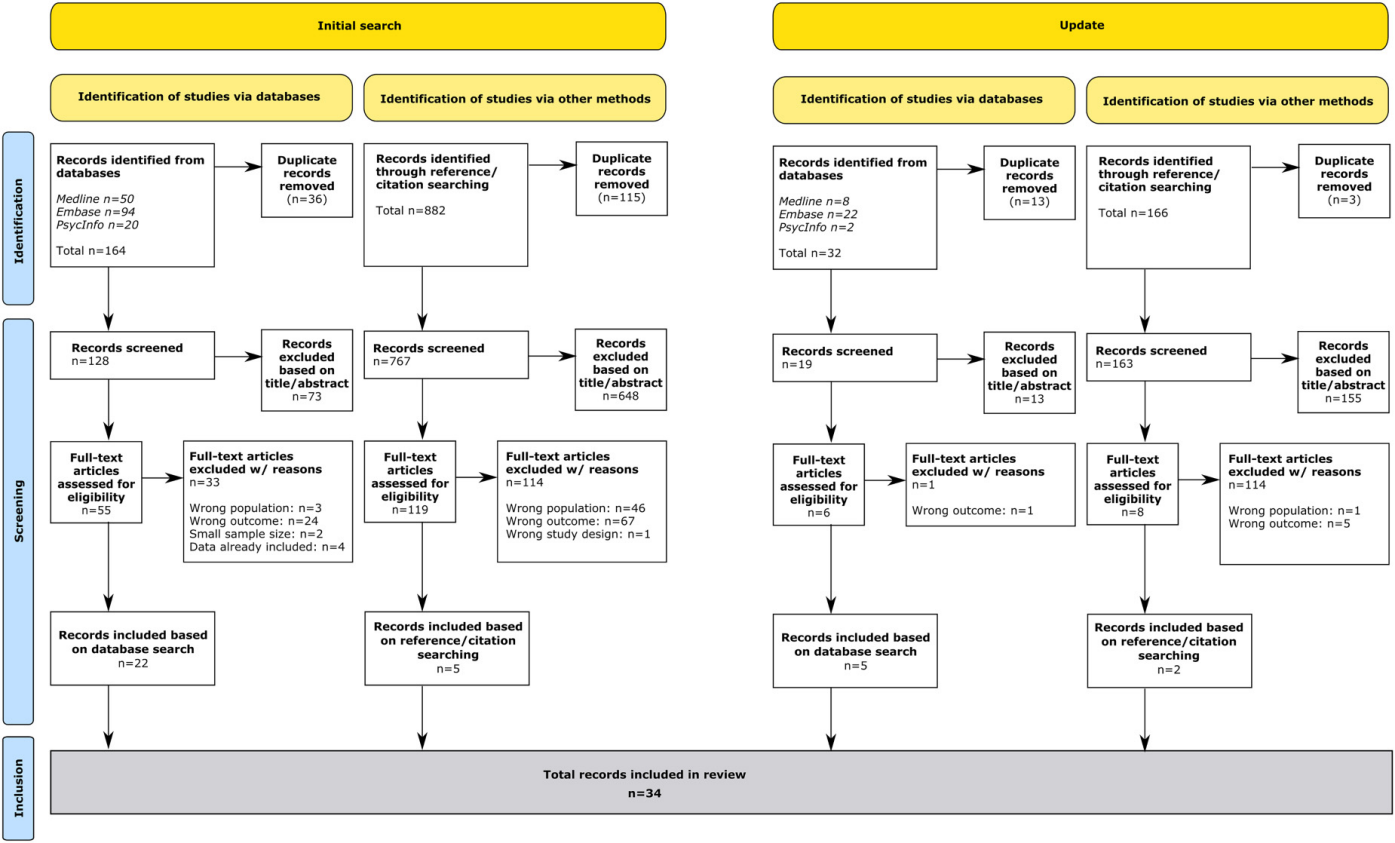
Prisma-Flowchart of study selection process.

A total of 196 records were identified across both the original and updated database searches. After removing duplicates and screening titles and abstracts, 61 articles underwent full-text review. Additionally, 1048 records were identified through reference and citation searching, of which 127 were screened at the full-text level. Ultimately, 34 records were included, reporting on 33 studies involving over 1,200,000 PwPD. The selection process is displayed in the PRISMA flow chart shown in Figure 1. Six authors of studies meeting the eligibility criteria but lacking essential information were contacted; three responded, but only one provided additional details. The places of death analysed by the identified studies included: home, long-term care facilities (hereafter nursing home), hospital, and hospice.

### Study characteristics

The studies, published between 2006 and 2025, included journal articles (n = 25),^18,25,28–51^ conference abstracts (n = 6),^52–57^ dissertations (n = 2),^58,59^ and one manuscript on an open-access publishing platform.^51^ The publishing journals primarily focused on palliative care (n = 9), neurology/neuroscience (n = 9), multidisciplinary medicine/public health (n = 3), geriatrics (n = 2), and other disciplines (n = 2). Geographically, the studies originated from Europe (n = 16), North America (n = 10), Asia (n = 3), South America (n = 2), and Australia (n = 1), with one study reporting international data from 11 countries. All publications were available in English except for one German dissertation.

### Quality assessment

Overall, the quality of the studies was considered to be high: 30 of the 33 studies were of high quality, achieving a summary score of 70% or more, while 3 studies were classified as having moderate quality, with scores between 50% and 70%. Both reviewers assigned the same overall score to 13 studies. Mean discrepancy in the overall scores was very low (0.02). Across studies, the main potential sources of bias were most likely related to incomplete reporting for example with regard to research question, study design and analytic methods.

### Distribution of place of death

The distribution of places of death among the identified studies varied considerably with the proportion of home deaths for PwPD ranging from 5.9% (Canada) to 73.0% (Mexico).^25,31,32,34–46^^,51,54,56–60^ Nursing homes were recorded as the place of death for 3.6% (Brazil) to 71.3% (New Zealand) of PwPD, while in a French cohort of nursing home residents, 77.1% died in such facilities.^25,31–34^^,36–49,51,52,54,57–60^ The proportion of hospital deaths ranged from 12.5% (USA) to 74.4% (South Korea).^18,25,29–34^^,36–42,44–47,49,51–59^ Only three studies specifically examined palliative care units as a place of death, reporting percentages from 0.6% (Germany) to 17.4% (Canada).^32,56,58^ The percentage of hospice death varied from 0.0% (Germany/UK) to 8.7% (Germany) for PwPD.^32,34,37,39,41–43^^,46,47,54,58,59^

### Meta-analysis

Meta-analyses could be conducted only for associations with hospital death, and statistically significant associations were identified for three factors: gender, age, and marital status.

**Figure 2.**
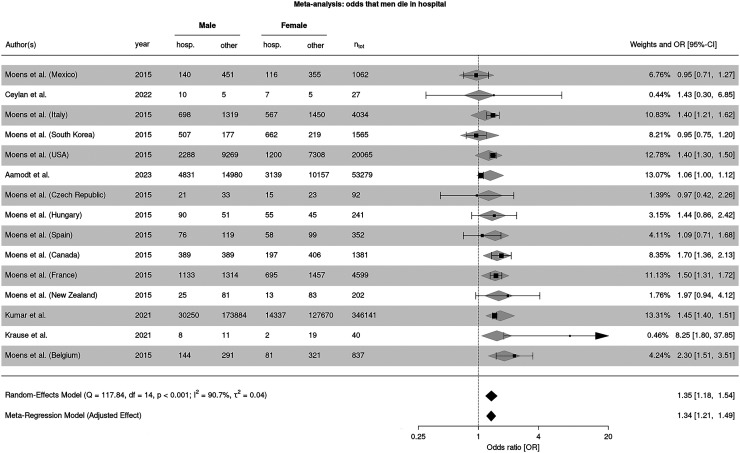
Forest plot of odds ratios (OR) and 95% confidence intervals (CI) for the association between male sex and hospital death among PwPD. Individual study estimates are shown together with pooled results from a random-effects meta-analysis and a meta-regression model.

The meta-analysis using both models revealed higher odds of hospital death for men with PD compared to women (see Figure 2). In a mixed-effects model using long-term beds and hospital beds as moderators the between-study heterogeneity variance was estimated at τ^2^ = .0016 (95% CI: <.001–.03), with an I^2^ value of 11% (95% CI: <.01–72.4%). An illustration is shown in the Supplement.

**Figure 3.**
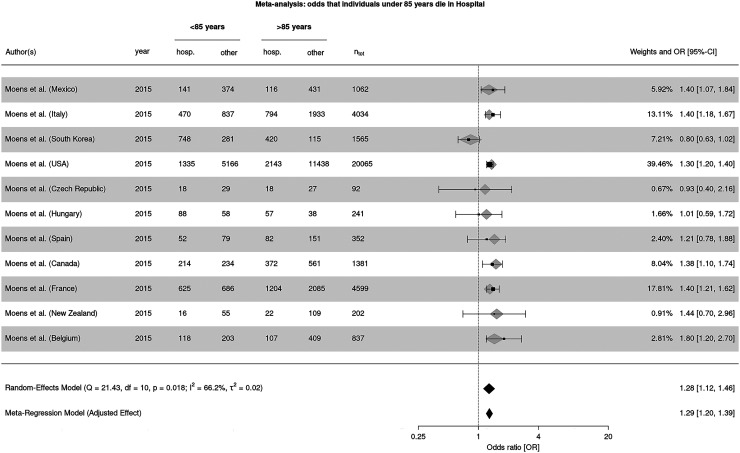
Forest plot of odds ratios (OR) and 95% confidence intervals (CI) for the association between age under 85 years and hospital death among PwPD. Individual study estimates are shown together with pooled results from a random-effects meta-analysis and a meta-regression model.

For PwPD at younger ages (<85 years), mixed-effects model with long-term beds and hospital beds as moderators the between-study heterogeneity variance was estimated at τ^2^ = 0.02 (95% CI: .01-.26), with an I^2^ value of 78% (95% CI: 46.9–97.7%) (see Figure 3).

**Figure 4.**
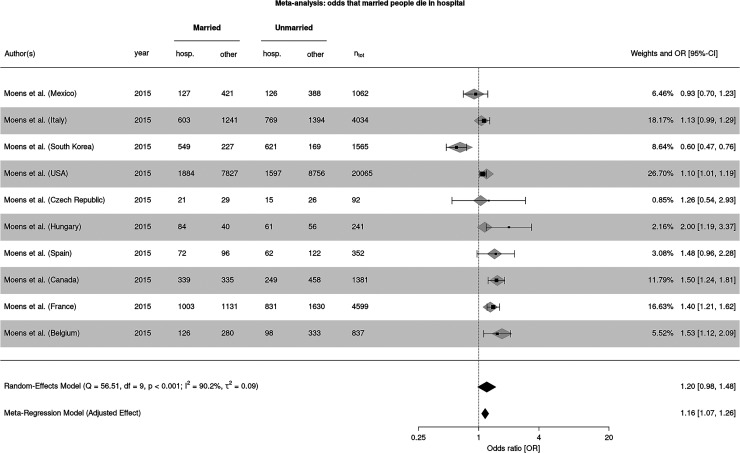
Forest plot of odds ratios (OR) and 95% confidence intervals (CI) for the association between being married and hospital death among PwPD. Individual study estimates are shown together with pooled results from a random-effects meta-analysis and a meta-regression model.

Additionally, the meta-regression model identified an increased likelihood of hospital death among married PwPD (see Figure 4). In the model using long-term beds and hospital beds as moderators the between-study heterogeneity variance was τ^2^ = .0043 (95% CI: <.001–.01), with an I^2^ value of 31% (95% CI: <.01–93.6%). Neither model demonstrated significant associations between hospital death and higher education or college education.

Neither model of the meta-analysis demonstrated significant associations between hospital death and urban versus rural residence (cf. Supplement).

### Narrative results

#### Home death

##### Individual factors

Two studies revealed a decrease in the likelihood of dying at home among men (study 1: OR = 0.86, 95% CI: 0.84–0.88^34^; study 2: OR = 0.16, 95% CI: 0.03–0.86^58^). Kumar et al.'s study did not demonstrate associations between different age groups and home death.^34^ However, the same study found that the odds of dying at home were almost doubled for individuals who were married (OR = 1.98, 95% CI: 1.93–2.03) and were also higher for PwPD with Black (OR = 1.32, 95% CI: 1.25–1.39), Hispanic (OR = 1.83, 95% CI: 1.75–1.92), and Asian ethnicity (OR = 1.48, 95% CI: 1.38–1.59).^34^ In this study, belonging to the Native American ethnicity group (OR = 1.19, 95% CI: 0.97–1.45) was not linked to home death. However, evidence indicated that college education raised the odds of home death (OR = 1.11, 95% CI: 1.09–1.14).^34^ Additionally, possessing a POLST (Physician Orders for Life-Sustaining Treatment) was associated with a higher likelihood of dying at home (OR = 1.76, 95% CI: 1.35–2.31).^37^

##### Illness-level factors

While cancer as a cause of death was associated with higher odds of dying at home (OR = 2.57, 95% CI: 1.46–4.53), PwPD dying from dementia had lower odds (OR = 0.68, 95% CI: 0.30–1.54). There were no significant results for the following causes of death: COPD (OR = 1.75, 95% CI: 0.69–4,49), heart diseases (OR = 1.34, 95% CI: 0.83–2.17), cerebrovascular diseases (OR = 0.85, 95% CI: 0.42–1.73), influenza/pneumonia (OR = 0.25, 95% CI: 0.06–1.11), accidents/falls (OR = 0.44, 95% CI: 0.17–1.18), and PD (OR = 1.02, 95% CI: 0.78–1.34).^44^

##### Service-level factors

In McKenzie et al.'s study, home death was positively associated with a longer duration of home care support (OR = 1.0007, 95% CI: 1.0004–1.0009), neurological outpatient care (OR = 1.91, 95% CI: 1.30–2.81) and outpatient palliative care (OR = 2.49, 95% CI: 1.30–2.81) in the last year of life.^37^

##### Environmental factors

One study identified an association between urban residence and an increased likelihood of dying at home (OR = 2.50, 95% CI: 2.43–2.59),^34^ while another study did not support this finding (OR = 0.78, 95% CI: 0.39–1.54).^45^ Additionally, three studies utilizing the CDC WONDER database identified time trends, with Weresh et al. analysing the longest time span.^34,43,47^ In their study, the odds of dying at home in 2022 were nearly 2.5 times higher compared to 2003 (OR = 2.46, 95% CI: 2.38–2.54).

#### Hospital death

##### Individual factors

Notably, the association between age and hospital death was examined through various methods and age categories in additional studies not suitable for meta-analysis, yielding inconclusive results. Consistent with the meta-analysis, two studies observed an increased risk of hospital death with younger age,^18,34^ while a third study observed a similar trend that did not reach statistical significance.^50^ Conversely, two other studies observed the opposite trend, with decreased odds of hospital death as age increased; however, effect sizes decreased in age categories above 75 years.^29,49^

Furthermore, two studies reported higher odds of hospital death for PwPD belonging to certain ethnic groups.^18,34^ These groups included Black (study 1: OR = 1.11, 95% CI: 1.00–1.24; study 2: OR = 1.77, 95% CI: 1.65–1.91), Hispanic (study 1: OR = 1.59, 95% CI: 1.33–1.91; study 2: OR = 1.44, 95% CI: 1.35–1.55), Asian (study 1: OR = 2.49, 95% CI: 2.10–2.96; study 2: OR = 2.09, 95% CI: 1.91–2.29), and Native American individuals (study 1: OR = 1.49, 95% CI: 1.05–2.10; study 2: OR = 1.67, 95% CI: 1.29–2.17).

Three studies investigated the association of socioeconomic status with hospital death in PD. Zwicker et al. found no associations with low (OR = 1.01, 95% CI: 0.85–1.20), middle (OR = 0.88, 95% CI: 0.74–1.05), high (OR = 0.98, 95% CI: 0.81–1.17), and highest income levels (OR = 0.95, 95% CI: 0.80–1.14) compared to those with the lowest income.^49^ Likewise, Leavy et al. identified a non-significant trend indicating that individuals from less advantaged groups may face a reduced risk of hospital death (OR = 0.86, 95% CI: 0.62–1.20).^50^ However, Aamodt et al. identified higher odds of hospital death for PwPD classified as socially deprived (OR = 1.24, 95% CI: 1.14–1.36).^18^

One study found that possessing a POLST was associated with a reduced likelihood of dying in hospital (OR = 0.45, 95% CI 0.29–0.70).^37^

##### Illness-level factors

Four studies investigated comorbidities or causes of death as factors associated with hospital death among PwPD. Zwicker et al. reported a higher likelihood of hospital death with an increasing number of active conditions (OR = 1.17, 95% CI: 1.15–1.22),^49^ while Aamodt et al. did not reveal an association with the Charlson Comorbidity Index (OR = 1.00, 95% CI: 0.99–1.01).^18^ One study identified frailty being associated with a threefold risk of hospital death (OR = 3.44, 96% CI: 2.24–5.26).^50^ Ceylan et al. found that chronic renal failure nearly doubled the odds of hospital death for PwPD (OR = 1.81, 95% CI: 1.10–2.98).^29^ In Tuck et al.'s study, Parkinson's disease as the cause of death was associated with a lower likelihood of hospital death (OR = 0.36, 95% CI: 0.25–0.52), whereas deaths attributed to accidents or falls (OR = 6.20, 95% CI: 2.91–13.20) and influenza or pneumonia (OR = 4.65, 95% CI: 1.77–12.21) were linked to higher odds of dying in a hospital.^44^ However, dying from chronic obstructive pulmonary disease (COPD) (OR = 1.41, 95% CI: 0.40–4.94), heart disease (OR = 1.32, 95% CI: 0.69–2.52), cerebrovascular disease (OR = 1.28, 95% CI: 0.52–3.12), dementia (OR = 0.44, 95% CI: 0.10–1.87), or cancer (OR = 0.28, 95% CI: 0.07–1.15) was not associated with hospital death.^44^

##### Service-level factors

Three studies identified that palliative care services were associated with a lower likelihood of hospital death (study 1: OR = 0.59, 95% CI: 0.41–0.87; study 2: OR = 0.24, 95% CI: 0.20–0.30; study 3: OR = 0.05, 95% CI: 0.02–0.15).^33,44,50^ Likewise, PwPD receiving palliative physician home visits (OR = 0.38, 95% CI: 0.34–0.43)^44^ and more family physician visits had lower odds of dying in hospitals (OR = 0.99, 95% CI: 0.89–0.99).^37^ Conversely, non-palliative home care (OR = 1.21, 95% CI: 1.04–1.42)^49^ as well as neurological outpatient care (OR = 1.37, 95% CI: 1.10–1.70), more hospitalisations (OR = 1.63, 95% CI: 1.36–1.95) and ICU admissions (OR = 3.79, 95% CI: 1,60–9.01) in the last year of life^37^ were linked to a higher likelihood of hospital death.

##### Environmental factors

Analysis of CDC WONDER data, referenced in three studies, indicated a sustained downward trend in hospital deaths from 2003 to 2022 in the United States (OR = 0.53, 95% CI: 0.51–0.55).^34,43,47^ In contrast, Ceylan et al. found no evidence of increased odds during the COVID-19 pandemic in Turkey (OR = 1.06, 95% CI: 0.35–3.18).^30^

#### Nursing home death

##### Individual factors

One study reported lower odds of nursing home death for men (OR = 0.96, 95% CI: 0.93–0.98),^34^ whereas a smaller study did not replicate this finding (OR = 0.36, 95% CI: 0.09–1.44).^58^ Kumar et al. identified significant associations between age groups over 64 years and increased likelihood of nursing home death.^34^ Additionally, the odds of dying in a nursing home were lower for individuals who were married (OR = 0.54, 95% CI: 0.53–0.56), had a college education (OR = 0.84, 95% CI: 0.82–0.86), or belonged to non-white populations, including Black (OR = 0.57, 95% CI: 0.54–0.60), Hispanic (OR = 0.42, 95% CI: 0.40–0.44), and Asian individuals (OR = 0.48, 95% CI: 0.44–0.52), with the exception of Native Americans (OR = 0.83, 95% CI: 0.68–1.01).^34^ Conversely, Tuck et al. found no association between having a POLST and the likelihood of nursing home death (OR = 0.97, 95% CI: 0.75–1.24).^44^

##### Illness-level factors

PwPD dying of PD were more likely to die in a nursing home (OR = 1.77, 95% CI: 1.37–2.28), whereas accidents as cause of death was significantly associated with lower odds of dying at home (OR = 0.26, 95% CI: 0.12–0.58). There were no indicative findings that the following causes of death were linked to a nursing home death: cancer (OR = 0.57, 95% CI: 0.32–1.02), COPD (OR = 0.45, 95% CI: 0.17–1.21), heart diseases (OR = 0.70, 95% CI: 0.44–1.12), cerebrovascular diseases (OR = 1.07, 95% CI: 0.56–2.03), dementia (OR = 1.86, 95% CI: 0.89–3.87) and influenza/pneumonia (OR = 0.45, 95% CI: 0.17–1.21).^44^

##### Service-level factors

In McKenzie et al.'s study, nursing home death was less likely among individuals receiving outpatient palliative care (OR = 0.59, 95% CI: 0.39–0.89) and neurologic outpatient care (OR = 0.50, 95% CI: 0.40–0.63) in the last year of life.^37^

##### Environmental factors

Furthermore, multiple studies drawing on the CDC WONDER database have highlighted temporal trends, with Weresh et al. examining the most extended period.^34,43,47^ Our analysis of the provided data found that the odds of dying in a nursing home in 2022 were 52% lower compared to 2003 (OR = 0.48, 95% CI: 0.46–0.49).^47^ Conversely, two studies provided conflicting evidence regarding the association between living in an urban environment and nursing home death. One study reported lower odds (OR = 0.68, 95% CI: 0.67–0.69),^31^ while the other found no significant association (OR = 1.12, 95% CI: 0.59–2.12).^45^

#### Hospice death

##### Individual factors

Two studies provided conflicting evidence regarding the association between male gender and hospice death. One study reported higher odds (OR = 1.23, 95% CI: 1.18–1.28),^34^ whereas the other found no significant association (OR = 6.14, 95% CI: 0.28–136.54).^58^ Kumar et al. identified a decreased likelihood of dying in hospice for age groups of 85 years and above compared to PwPD younger than 65 years (OR = 0.80, 95% CI: 0.71–0.90) but not for other age categories in between.^34^ Further, the authors found increased odds of dying in hospice for married individuals (OR = 1.24, 95% CI: 1.19–1.30).^34^ In the same study, PwPD with black (OR = 1.10, 95% CI: 1.003–1.20) or Hispanic ethnicity (OR = 1.12, 95% CI: 1.003–1.21) were slightly more likely to die in hospice compared to white PwPD but those with an Asian (OR = 0.60, 95% CI: 0.52–0.70) and Native American ethnicity (OR = 0.57, 95% CI: 0.73–0.88) were less likely to die in this setting.^34^ Lastly, no association between college education and hospice death was observed (OR = 1.03, 95% CI: 0.99–1.07).^34^

##### Illness-level factors

No study investigated illness-level factors associated with hospice death.

##### Service-level factors

Evidence from one study further suggests that likelihood of hospice death was substantially higher for those PwPD who received an inpatient (OR = 15.79, 95% CI: 8.64–28.84) or outpatient (OR = 5.73, 95% CI: 3.03–10.89) palliative care consultation or neurological outpatient care (OR = 3.12, 95% CI: 1.81–5.40) in the last year of life.^37^

##### Environmental factors

Data from the CDC WONDER database, used by four studies, demonstrate a clear time trend, with the odds of dying in hospice in 2022 being 40 times higher than in 2003 (OR = 40.67, 95% CI: 32.31–51.18).^34,43,47^ Moreover, Kumar et al. reported a higher likelihood of hospice death for individuals with urban residence (OR = 2.55, 95% CI: 2.42–2.69)^34^ Table 1.

**Table 1.** Overview of study characteristics.

First author (year)	Country of origin	Data source	Population	n (total)*	Mean/ median age**	Female (%)**	Ethnicity/racial composition (%)	PoD distribution	Factors studied
Aamodt (2023)^18^	USA	Administrative data	PD	53,279	83 (M)	43.1%	White: 89.5%	Hospital: 15%	Age, gender, ethnicity, social deprivation, comorbidities, residence
							Black 5.4%		
							Hispanic: 1.6%		
							Asian: 1.5%		
							North America native: 0.4%		
							Unknown/Other: 1.6%		
Akhtar (2025)^60^	USA	Administrative data	PD with cardiovascular disease-related death	138,151	Not given	42.9%	White: 87.6%	Home: 23.7%	None
							Black/African American: 5.1%	Nursing home: 43.3%	
							Hispanic: 4.6%		
							Asian/Pacific Islander: 2.3%		
							American Indian/Alaska Native: 0.3%		
Ceylan (2022/2023)^29,30^	Turkey	Medical records	Geriatric population	110	81 (Mdn)	54.6%	Not given	Hospital: 63.6%	Age, gender, chronic renal failure, COVID19-pandemic
Cohen (2006)^31^	Belgium	Administrative data	General population	252	Not given	Not given	Not given	Home: 16.3%	None
								Hospital: 34.1%	
								Nursing home: 49.6%	
Dasch (2018)^32^	Germany	Administrative data	Dementia	1646	Not given	Not given	Not given	Home: 16.9%	None
								Hospital: 35.1%	
								Nursing home: 47.4%	
								Hospice 0.0%	
								PCU: 0.6%	
Giffard (2023)^52^	France	Medical records	PDRD living in nursing home	109	87 (M)	Not given	Not given	Hospital: 22.0%	None
								Nursing home: 77.1%	
Grose (2020)^53^	UK	Medical records	PD (known to Movement Disorder Service)	Not given	Not given	Not given	Not given	Hospital: 59.0%	None
Hill (2020)^54^	UK	Medical records	PD	127	82,7 (M)	NA	Not given	Home: 25.1%	None
								Hospital: 48,8%	
								Nursing home: 19.7%	
Hobson (2018)^33^	UK	Medical records/death certificates	PD	166	80.7 (M)	44.0%	Not given	Hospital: 37,0%	None
Krause (2021)^58^	Germany	Administrative data	General population	23	83,2 (M)	39.1%	Not given	Home: 17.4%	None
								Hospital: 34,8%	
								Nursing home: 21.7%	
								Hospice 8.7%	
								PCU: 17.4%	
Kumar (2021)^34^	USA	Administrative data	PD	346,141	NA	41.0%	White: 93.7%	Home: 27.6%	Age, gender, marital status, ethnicity/race, education, residence
							Hispanic: 4.6%	Hospital: 12.9%	
							Black: 4.0%	Nursing home: 45.9%	
							Native Americans: 0.3%		
								Hospice: 5.6%	
							Asian/Pacific Islander: 2.0%		
Leavy (2025)^50^	Sweden	Administrative data	PD	922	80.2 (7.0)	37.0%	Not given	Hospital: 30.8%	Age, Sex, socioeconomic status, frailty, specialist palliative care
Lin (2024)^35^	Taiwan	Administrative data	General population	892	Not given	Not given	Not given	Home 54.8%	None
Mai (2018)^36^	South Korea	Administrative data	Geriatric population	20,161	Not given	Not given	Not given	Home: 24.1%	None
								Hospital: 64.3%	
								Nursing home: 5.9%	
Mappilakkandy (2017)^55^	UK	Medical records/death certificates	PD (known to service)	40	Not given	Not given	Not given	Hospital: 57%	None
McKenzie (2022)^37^	Canada	Administrative data	Movement disorders	1226	Not given	40.0%	Not given	Home: 7.1%	Residence, dementia, healthcare utilisation
								Hospital: 46.2%	
								Nursing home: 37.2%	
Miyasaki (2012)^56^	Canada	Medical records	PD known to palliative care service	100	Not given	Not given	Not given	Home: 72.7%	None
								Hospital: 22.7%	
								PCU: 4.5%	
Moens (2015)^25^	international	Administrative data	PD	34,430	Not given	41.3% (Czech Republic) – 56.3% (South Korea)	41.3% (Czech Republic) – 56.3% (South Korea)	Home: 5.9 (New Zealand/Canada) – 73.0 (Mexico)	Age, gender, education, martial status, residence, healthcare system factors
								Hospital: 17.3% (USA) – 74.7% (South Korea)	
								Nursing home: 4.5% (South Korea) – 71.3% (New Zealand)	
Morris (2019)^59^	UK	Bereavement survey	PDRD	2029	Not given	41.0%	White: 93%	Home: 13%	None
								Hospital: 34%	
								Nursing home: 51%	
								Hospice: 2%	
Moscovich (2014)^57^	Brazil	Not given	PDRD	205	76,8 (M)	38.5%	Not given	Home: 26.8%	None
								Hospital: 68.9%	
								Nursing home: 4.5%	
Moscovich (2017)^38^	Brazil	Clinical data/death certificates	PDRD	138	77,9 (M)	45.1%	Not given	Home: 21.0%	None
								Hospital: 72.2%	
								Nursing home: 3.6%	
Nicholas (2021)^39^	UK	Registry data	PD	579	79,7 (M)	41.6%	Not given	Home: 11.6%	None
								Hospital: 35.3%	
								Nursing home: 52.9%	
								Hospice: 0.3%	
Shi (2021)^40^	UK	Clinical data/death certificates	PDRD	277	81,9 (M)	35.0%	Not given	Home: 13.7%	None
								Hospital: 48.3%	
								Nursing home: 37.9%	
Sleeman (2013)^41^	UK	Administrative data	PD	125,242	81,5 (M)	43.0%	Not given	Home: 9.7%	None
								Hospital: 43.4%	
								Nursing home: 46.3%	
								Hospice: 0.6%	
Snell (2009)^42^	UK	Administrative data/clinical data	PD	143	81,5 (M)	47.6%	Not given	Home: 9.0%	None
								Hospital: 55.0%	
								Nursing home: 36.0	
								Hospice: 0.0%	
Sokhal (2024)^51^	USA	Administrative data	PD	515,884	Not given	39.2%	White: 91.5%	Home: 28.3%	None
							Black/African American: 5.2%	Hospital: 12.7%	
								Nursing home: 45.7%	
							Asian/Pacific Islander: 2.9%		
							American Indian/Alaska Native: 0.3%		
Tharwani (2024)^43^	USA	Administrative data	PD	830,176	NA	41.3%	White: 84.8%	Home: 25.6%	Time trend
							Hispanic/Latino: 6.5%		
							Black/African American: 5.2%		
							Asian/Pacific Islander: 3.0%		
							American Indian/Alaska Native: 0.4%		
								Nursing home: 44.3%	
								Hospice: 4.8%	
Tuck (2015)^44^	USA	Death certificates/Registry data	PD	58,034	83 (Mdn)	32.9%	Hispanic/Latino 1.2%	Home: 31.5%	POLST
							African American: 0.5%	Hospital: 12.5%	
								Nursing home: 52.3%	
							American Indian/Alaska Native 0.5%		
							Asian/Native Hawaiian/Pacific Islander 1.6%		
							Caucasian 96.9%		
							More than one race marked 0.3%		
							Other race not listed: 0.3%		
Walker (2013)^45^	UK	Clinical data	PD	236	82,8	54.7%	Not given	Home: 23.7%	None
								Hospital: 46.6%	
								Nursing Home: 25.0%	
Weresh (2025)^47^	USA	Administrative data	PD	947,272	Not given	40.9%	Non-Hispanic White: 88.0%	Home: 27.1%	Time trend
								Hospital: 16.7%	
							Hispanic/Latino: 4.9%	Nursing home: 42.7%	
							Black/African American: 4.3%	Hospice: 5.2%	
							American Indian/Alaskan: 0.3%		
Wilson (2024)^46^	UK	Administrative data	PDRD	56,790	Not given	Not given	Not given	Home: 19.2%	none
								Hospital: 39.2%	
								Nursing home: 38.8%	
								Hospice: 2.0%	
Xu (2021)^48^	Australia	Administrative data	PD with DBS	1849	72,9 (M)	Not given	Not given	Nursing home: 68.8%	None
Zwicker (2022)^49^	Canada	Administrative data	PD	291,276	77 (M)	Not given	Not given	Hospital: 36%	Age, gender, Residence, healthcare use, income, number of active conditions
								Nursing home: 39%	

*PD level, if possible.

*PD level only.

M = mean, Mdn = Median, PCU = palliative care unit, PD = Parkinson's disease, PDRD = Parkinson's disease and related disorders, PoD = place of death.

## Discussion

### Main findings

Drawing on data from 33 original studies, our systematic review identified two key findings: (i) the distribution of place of death among PwPD varies considerably across countries, and (ii) pooled data suggests that male gender, being married, and younger age increase the likelihood of hospital death.

A striking finding is the significant variation in place of death among PwPD across studies conducted on five continents. In line with previous research on other populations, these differences may stem from disparities in healthcare resources and country-specific policies shaping end-of-life care as well as cultural factors.^61–63^ However, research on the place of death for PwPD remains limited, with existing evidence focusing on hospital deaths. In particular, specialised end-of-life care settings, such as palliative care units and hospices, are underrepresented, underscoring the need for further research into their role in supporting PwPD at the end of life.

### Gender and social factors

Although a minority of PwPD wishes to die in a hospital,^14–16^ our meta-analysis revealed a higher likelihood of hospital deaths among men, younger individuals and those who are married. While gender differences in home death preferences are not well-documented, men may face higher hospitalisation rates due to sex-specific comorbidities for PwPD, and differences in healthcare-seeking behaviours, societal roles, or caregiving support.^16,64–67^ Women with PD, for example, are more likely to transition to paid caregiving and are more likely to utilise advanced nursing care,^64,68^ whereas they exhibit lower rates of direct physician contact and are less likely to receive intensive care at the end of life.^18,64^ In contrast, caregivers of men with PD report experiencing greater strain.^65^ This possibly suggests less robust support networks for men and a higher likelihood of receiving aggressive medical interventions at the end of life compared to women. Further research is needed to clarify these patterns and inform gender-sensitive care strategies.

Beyond gender differences, social and caregiving dynamics also play a crucial role in determining the place of death. Marital status, in particular has been shown to influence end-of-life care, with married individuals having an increased likelihood of dying at home in non-PD populations including neurodegenerative diseases, possibly due to caregiving by live-in family members.^62,69–71^ By contrast, our review found that married PwPD were more likely to die in hospital even though informal caregiving is common in advanced stages.^16^ This reversal may reflect the long and progressive trajectory of PD. As disability accumulates over many years, the sustained caregiving burden may ultimately exceed a spouse's capacity to continue providing care at home until death.^72,73^ PwPD frequently die from complications of the disease, such as infections,^74^ which, although medically predictable, can nevertheless be perceived by families as unexpected events. Such circumstances may precipitate a breakdown of family caregiving capacity resulting in hospital admission. This care challenge may appear less pronounced when formal caregivers are involved underscoring the need for targeted support to alleviate the strain on spousal caregivers. Interventions such as caregiver training, respite care, and expanded access to formal caregiving services could help sustain home-based care and better align with patients’ end-of-life preferences. Additionally, early advanced care planning (ACP) and family education may be essential in proactively addressing caregiving challenges, ensuring continuity of care and improving PwPD's quality of life.^4,75,76^

As observed in other neurodegenerative diseases, our meta-analyses further identified younger age as a potential risk factor for hospital death in PD.^71,77,78^ Various explanations for this association are plausible, suggesting that the effect is likely multifactorial. First, a significant proportion of PwPD die from causes unrelated to PD, which become more prevalent with increasing age,^38,79^ possibly shifting end-of-life care patterns in older patients. Safarpour et al. found that community-dwelling PwPD were generally younger than those residing in long-term care facilities.^80^ This age difference may be relevant when interpreting place of death-patterns as a higher number of elderly PwPD may receive more comprehensive formal care enabling out-of-hospital death. Moreover, younger people are less likely to engage in end-of-life care planning^81^ and may opt for more life-prolonging interventions,^82^ which may result in more aggressive end-of-life care strategies in young PwPD requiring hospital care. Ultimately, there is an urgent need for further research in this area to pinpoint the exact causes of unnecessary/unwanted hospital admissions at the end of life and develop targeted interventions. Integrating hypothetical yet typical disease trajectories into ACP or serious illness conversations may help clarify patient goals and enhance patient-centred care mitigating the risk of hospital death.

We identified lower-quality evidence suggesting that PwPD of non-white ethnicity may be associated with a higher probability of hospital death. Systematic reviews have previously demonstrated an increased likelihood of hospital encounters toward the end of life among non-white individuals in populations with cancer and heart failure.^83,84^ Throughout the disease trajectory, ethnic minority groups face significant disparities in access to care, delivery of services, and receipt of medical, surgical, rehabilitative, and palliative therapies.^85^ These challenges are further exacerbated by the underrepresentation of ethnic minorities in PD research.^85^ Our findings highlight the urgent need for researchers to explore inequalities in PD end-of-life care and call on healthcare providers to reflect on potential biases while prioritising culturally sensitive and individualised approaches to care. Additionally, we aim to raise awareness among organisations and policymakers to address systemic inequities and advocate for structural reforms to reduce disparities in end-of-life care for PwPD.

### Palliative care and system-level implications

Our findings also indicate that the receipt of palliative care is associated with a reduced likelihood of hospital death. This supports existing literature, which highlights that palliative care involvement increases the probability of dying in an out-of-hospital setting.^86,87^ However, despite similarities in the prevalence of palliative care-related needs between cancer and non-cancer patients, including those with PwPD,^88^ non-cancer populations remain disadvantaged in their access to palliative care.^89–91^ Expanding access to palliative care services has the potential to align with the preferences of PwPD to avoid hospital deaths^14–16^ and enhance patient and caregiver outcomes.^92^

Population-level mortality data have indicated shifts in place-of-death distributions during the COVID-19 pandemic, including increases in hospital and home deaths.^93,94^ However, such patterns were not evident in the single study examining this association. In an Italian study, PD itself did not appear to increase the risk of COVID-19–related hospitalisation, whereas parkinsonism was identified as an independent risk factor, likely reflecting greater disease severity, higher care dependence, and increased exposure in high-risk care settings.^95^ Post-pandemic developments also remain underexplored, as available time-trend studies extended at most to 2022. Further research is required to clarify place-of-death patterns in PD during the pandemic and their potential longer-term implications.

### Limitations

Our study's focus on meta-analytic findings provides a robust, evidence-based synthesis of factors associated with place of death of PwPD. However, this strength is accompanied by certain limitations. Although outcome definitions were generally consistent across studies, incomplete and variable reporting limited the availability of data for certain places of death and precluded meta-analysis for all outcomes. Moreover, the variability in place-of-death trends across the included countries may influence the generalisability of our results. Although grey literature was not systematically searched, the primary outcomes of interest in the included studies were largely derived from administrative data. Lastly, since the evidence is predominantly drawn from high-income countries, the applicability of our findings to low-resource settings remains constrained, emphasising the need for further research in diverse healthcare contexts.

## Conclusion

This systematic review identifies key factors associated with hospital death in PwPD, including gender, age, and marital status, which may guide actions to mitigate risks. These findings can assist clinicians and policymakers in developing interventions that promote goal-concordant care and potentially reduce hospital deaths, including through improved support for informal caregivers, timely access to palliative care services and better alignment of care with patient preferences. However, further research is needed to establish robust evidence on the factors that influence the place of death, as well as the facilitators and barriers to achieving preferred end-of-life settings across diverse healthcare systems.

## Supplemental Material

sj-docx-1-pkn-10.1177_1877718X261431105 - Supplemental material for Place of death in Parkinson's disease: A systematic review and meta-analysis of associated factors Supplemental material, sj-docx-1-pkn-10.1177_1877718X261431105 for Place of death in Parkinson's disease: A systematic review and meta-analysis of associated factors by Anna J Pedrosa, Florian Kuschel, Hanna K Fischer and David J Pedrosa in Journal of Parkinson's Disease
