# Supplementary material for: Place of death in Parkinson's disease: A systematic review and meta-analysis of associated factors
Source: J Parkinsons Dis. 2026 Apr 17:1877718X261431105. Online ahead of print. doi: 10.1177/1877718X261431105 (PMC13435204; doi:10.1177/1877718X261431105)
Supplement: Supplementary file 1 [file sj-docx-1-pkn-10.1177_1877718X261431105.docx]

**Search Strategy**

Medline (OVID interface)

1) Parkinson Disease/
2) Parkinson$.ti,ab.
3) 1) or 2)
4) (place adj2 death).ti,ab.
5) (location adj2 death).ti,ab.
6) (site adj2 death).ti,ab.
7) (care adj2 death).ti,ab.
8) (home adj2 death).ti,ab.
9) (hospital adj2 death).ti,ab.
10) (hospice adj2 death).ti,ab.
11) (institution? adj2 death).ti,ab.
12) 4) or 5) or 6) or 7) or 8) or 9) or 10) or 11)
13) 12) and 3)

EMBASE

1) ‚Parkinson disease‘
2) Parkinson?:ti,ab
3) 1) or 2)
4) ‚location of death‘
5) ‚place of death‘
6) (place NEAR/2 death):ti,ab
7) (location NEAR/2 death):ti,ab
8) (site NEAR/2 death):ti,ab
9) (care NEAR/2 death):ti,ab
10) (home NEAR/2 death):ti,ab
11) (hospital NEAR/2 death):ti,ab
12) (hospice NEAR/2 death):ti,ab
13) (institution? NEAR/2 death):ti,ab
14) 4) or 5) or 6) or 7) or 8) or 9) or 10) or 11) or 12) or 13)
15) 14) and 3)

APA PsycInfo

TI (parkinson*) OR AB (parkinson*) OR MA(parkinson's disease)

AND

TI(place N2 death OR location N2 death OR site N2 death OR care N2 OR home N2 death OR hospital N2 death OR hospice N2 death OR institution* N2 death) OR AB(place N2 death OR location N2 death OR site N2 death OR care N2 death OR home N2 death OR hospital N2 death OR hospice N2 death OR institution* N2 death

**Results Meta-Analyses**
